# Design and Evaluation of an Eye Mountable AutoDALK Robot for Deep Anterior Lamellar Keratoplasty

**DOI:** 10.3390/mi15060788

**Published:** 2024-06-15

**Authors:** Justin D. Opfermann, Yaning Wang, James Kaluna, Kensei Suzuki, William Gensheimer, Axel Krieger, Jin U. Kang

**Affiliations:** 1 Department of Mechanical Engineering, Johns Hopkins University, Baltimore, MD 21218, USA; jkaluna1@jhu.edu (J.K.); ksuzuki6@jh.edu (K.S.); axel@jhu.edu (A.K.); 2 Laboratory for Computational Sensing and Robotics, Johns Hopkins University, Baltimore, MD 21218, USA; ywang511@jhu.edu (Y.W.); jkang@jhu.edu (J.U.K.); 3 Department of Electrical and Computer Engineering, Johns Hopkins University, Baltimore, MD 21218, USA; 4 Ophthalmology Section, White River Junction Veterans Affairs Medical Center, White River Junction, VT 05009, USA; william.gensheimer@va.gov; 5 Ophthalmology Section, Dartmouth—Hitchcock Medical Center, Lebanon, NH 03766, USA

**Keywords:** micro-robotics, deep anterior lamellar keratoplasty, optical coherence tomography, piezoelectric, body mountable robotics

## Abstract

Partial-thickness corneal transplants using a deep anterior lamellar keratoplasty (DALK) approach has demonstrated better patient outcomes than a full-thickness cornea transplant. However, despite better clinical outcomes from the DALK procedure, adoption of the technique has been limited because the accurate insertion of the needle into the deep stroma remains technically challenging. In this work, we present a novel hands-free eye mountable robot for automatic needle placement in the cornea, AutoDALK, that has the potential to simplify this critical step in the DALK procedure. The system integrates dual light-weight linear piezo motors, an OCT A-scan distance sensor, and a vacuum trephine-inspired design to enable the safe, consistent, and controllable insertion of a needle into the cornea for the pneumodissection of the anterior cornea from the deep posterior cornea and Descemet’s membrane. AutoDALK was designed with feedback from expert corneal surgeons and performance was evaluated by finite element analysis simulation, benchtop testing, and ex vivo experiments to demonstrate the feasibility of the system for clinical applications. The mean open-loop positional deviation was 9.39 µm, while the system repeatability and accuracy were 39.48 µm and 43.18 µm, respectively. The maximum combined thrust of the system was found to be 1.72 N, which exceeds the clinical penetration force of the cornea. In a head-to-head ex vivo comparison against an expert surgeon using a freehand approach, AutoDALK achieved more consistent needle depth, which resulted in fewer perforations of Descemet’s membrane and significantly deeper pneumodissection of the stromal tissue. The results of this study indicate that robotic needle insertion has the potential to simplify the most challenging task of the DALK procedure, enable more consistent surgical outcomes for patients, and standardize partial-thickness corneal transplants as the gold standard of care if demonstrated to be more safe and more effective than penetrating keratoplasty.

## 1. Introduction

The cornea is an avascular tissue that plays an important role in the protection and function of the eye [1]. Structurally, the cornea is composed of five layers that include the epithelium, Bowman’s layer, stroma, Descement’s membrane, and endothelium [2]. The cornea provides up to two-thirds of the refractive power of the eye [3], so the integrity and health of these tissues play an integral role in a patient’s visual quality. For instance, corneal degenerations like keratoconus that cause thinning and weakening of the tissue can result in decreased vision [4]. Corneal opacities can also cause visual impairment and blindness [5]. An estimated 5.5 million people worldwide are bilaterally blind and an additional 6.2 million people unilaterally blind due to corneal opacities resulting from infectious keratitis, trachoma, onchocerciasis, and ocular trauma [6].

One surgical option for patients with corneal opacity is a full-thickness corneal transplant called a penetrating keratoplasty (PK). During this surgery, an ophthalmic surgeon replaces the full thickness of the host cornea with a donor graft including the endothelium [7]. Unfortunately, post-operative complications after PK include graft rejection [8], with up to 50% of rejections resulting from endothelial rejection [9]. Due to the risk of endothelial graft rejection, less invasive techniques that preserve the host endothelium such as deep anterior lamellar keratoplasty (DALK) [10] are also a surgical option for corneal opacities that do not involve the posterior cornea or Descemet’s membrane. In DALK surgery using the big bubble technique, a needle is inserted into the cornea to pneumodissect the anterior stromal tissue from the deep posterior stromal tissue and Descemet’s membrane (DM) [11,12], leaving the host DM and endothelium intact. The DALK technique reduces rates of endothelial rejection [9] and lessens the incidence of intraoperative complications [13]. When approximately 90% of the stromal tissue is dissected, there is no difference in corrected distance visual acuity between the DALK and PK procedure [14,15]. A report by the American Academy of Ophthalmology found that DALK is superior to PK in preserving endothelial cell density, may simplify long-term management of the eye, and is recommended as a surgical option for patients whose endothelium is not compromised [15]. 

**Figure 1:**
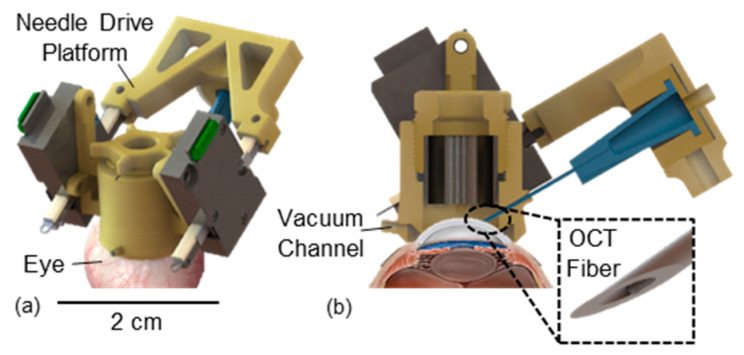
Isometric (**a**) and cross-sectional view (**b**) of the AutoDALK robot mounted on an eye. The 25 G needle with the OCT fiber is shown in the dashed box. A 2 cm scale bar is shown.

Despite the advantages of DALK, adoption is limited due to the technical difficulty of the procedure. Advances in technique such as big bubble dissection and ophthalmic viscosurgical devices help the surgeon reliably dissect layers of stromal tissue [16,17], but the accurate positioning of the needle up to 90% of stromal depth remains a major hurdle. DM perforation rates are reported between 31.8–60% during a surgeon’s first 10 grafts and remains as high as 11.7% despite years of experience [18,19]. There is a clear clinical need for a robotic needle placement system to standardize the DALK procedure irrespective of surgeon background or experience. In this work, we will present the design and evaluation of one such system to enable automatic needle placement in the cornea, known as AutoDALK (Figure 1).

## 2. Previous Works

Robotic systems to remove surgical tremors during needle insertion are one solution to improve ocular surgery. In 1999, Taylor et al. first introduced the steady-hand robotic system for microsurgery, which was modified by Mitchell et al. to be used in retinal surgery [20,21]. While using a force/torque input collected from the manual use of a surgical tool in the operating field, the robot simultaneously supports the surgeon’s arm while allowing voluntary motion and suppressing surgical tremor. With this strategy, the steady-hand robot provides a more direct coupling to the human’s natural kinesthetic sense as compared to teleoperated systems for ocular surgeries such as Preceyes [22]. However, these systems are large, rigidly attached to the surgical bed, and have high inertia with a large range motion, which can be problematic to account for if the patient makes sudden and unexpected movements, such as during ophthalmic surgery when a patient is sedated instead of anesthetized [23]. 

To enable robot integration with a surgical theater, a patient mounted robot was introduced by Nasseri et al. for intraocular micromanipulation [24]. The system mounts to the patient’s skull and uses a hybrid serial–parallel mechanism for micro-scale motion. While this strategy accounts for unexpected motions of the patient, the system is teleoperated, which disassociates the surgeon from the surgical field and removes tactile feedback, which is critical for the DALK procedure. Alternatively, two groups have introduced hand-held devices that provide needle motion compensation. The Micron [25] implements a six-degrees-of-freedom parallel mechanism to actively adjust the needle to compensate for hand motions, while Huang et al. used optical coherence tomography (OCT) to maintain a constant distance between a needle and target [26]. While both technologies enable tactile feedback with the surgeon in the loop, final depth control is manual and dependent on the surgeon’s skill and experience. To overcome variations in surgeon skill, Draelos et al. have created a robotic DALK system that uses B-scan OCT to generate depth profiles of the cornea for autonomous needle placement [27]. However, like the robotic systems previously discussed, the robot is large, requires intraoperative calibration between the OCT and robot to function, and cannot compensate for unexpected patient motions. 

To overcome the above limitations, our team previously described a robotic DALK system using OCT-A scan to generate one-dimensional depth measurements of the cornea and enable precise needle insertion with DM pneumodissection [28]. While the tool proved to be more consistent and accurate at placing a needle within 100 µm of the DM, the robot was based on a linear DC motor, which was heavy, cumbersome, and required the operator to manually balance during use. To overcome the weight limitations of this first design, the robot was miniaturized with a novel wax-based piezo stack that was light enough to mount to the eye [29]. We demonstrated that the actuator could repeatably reach 90% of the stromal thickness while under OCT guidance; however, the needle’s wax mechanism required more than 3 h for tissue penetration, making it impractical for ex vivo or in vivo studies.

In this paper, we describe the design and evaluation of a novel AutoDALK system (Figure 1) that to our knowledge represents the first robotic system capable of performing automatic DALK needle insertion to prescribed depths in the cornea at clinically acceptable speeds, and in a form factor small and light enough to be eye mountable. Our specific contributions of this paper are as follows: (1) to present the design of the eye mountable robot inspired by clinical requirements; (2) a finite element analysis (FEA) to demonstrate that the system is robust to expected clinical forces; (3) the integration of a common swept source OCT system for depth measurement; (4) the evaluation of the robot’s mechanical performance as defined by maximum penetration force and positional accuracy measured to international standards; finally, (5) a comparison study against an expert surgeon to evaluate the clinical performance of the AutoDALK system to pneumodissect ex vivo porcine eyes for the DALK procedure. 

## 3. Materials and Methods

### 3.1. Clinical Requirements for the AutoDALK Robot

The design of an eye mountable AutoDALK robot is a non-trivial task. To ensure that the performance of the final system would be appropriate for clinical use, we interviewed key opinion leaders and performed a literature review based on recommendations from the clinical interviews to determine the appropriate clinical requirements with associated engineering specifications and ideal performance metrics, as shown in Table 1 below. Engineering specifications were taken from the reported averages in each reference.

An eye mountable design was desired for the DALK procedure as this approach mechanically couples the robot to the eye, which maintains the relative needle position without the need for intraoperative registration. However, by being eye mountable, the system’s mass and footprint become crucial so that the device does not detach from the eye. To ensure clinically acceptable performance, we chose to attach the robot to the eye by using a vacuum syringe (Figure 1b) inspired by clinically approved ocular devices. To determine the engineering specifications in Table 1, we first limited the device size to 24 mm × 24 mm × 24 mm so that it did not exceed the average diameter of the human eye [30]. Next, we measured the torque to separate a commercial vacuum-assisted trephine (Moria, Massy, France) from ex vivo rabbit cornea to be τ=840 gmm. Using Equation (1) with r=12 mm, θ=90°, and $factor\;of\;safety\left(FOS\right)=2$, the allowable mass was m<35 g.

(1) $${mass}_{max}=\frac{\tau }{r\times FOS\sin\left(\theta \right)}$$

To consistently pierce the cornea, the AutoDALK must also generate more than 0.518 N of force [31], have a positioning resolution better than 1 µm so that it does not pierce the DM, which averages 7.49–11.97 μm thick [32], and must be compatible with small-gauge needles. Lastly, the insertion speed was set up to 5 mm/s to be consistent with needle penetration tests in ex vivo tissue [33]. 

### 3.2. AutoDALK Design

**Figure 2:**
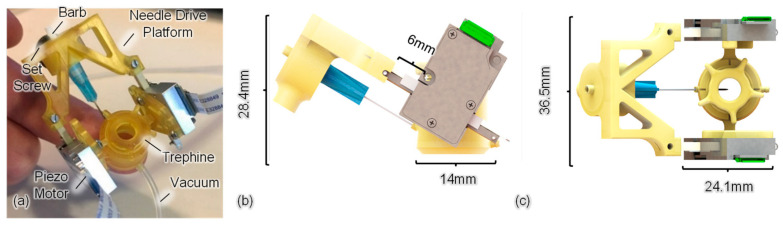
Prototype of the piezo-based eye mountable AutoDALK system with key features identified (**a**). A three-dimensional rendering of the front (**b**) and top view (**c**) with key dimensions labelled.

The AutoDALK system was designed using Solidworks software 2022 SP5 (Dassault Systèmes, Waltham, MA, USA) and prototyped using a microArch 3D printer with 40 µm resolution (Boston Micro Fabrication, Maynard, MA, USA). The system was constructed from a non-porous biocompatible material that is ISO 10993 [34] certified for acute skin contact and can be disinfected by using enzymatic cleaner with an alcohol scrub or sterilized with low-temperature sterilization for in vivo experiments. As illustrated in Figure 2, the AutoDALK system integrates a trephine, quick-attach needle drive platform, and two piezo-based motors. The main trephine body includes a vacuum chamber and port, which is connected to a commercial spring-loaded syringe. When placed on the eye, the syringe creates a vacuum on the underside of the trephine that fixes the trephine to the eye with a vacuum pressure that does not exceed that of regulated medical devices. Linear piezo electric motors are used to actuate the needle and are rigidly fixed to the trephine with screws. Piezo motors are used for the linear actuation due to their light weight and relatively high force generation. The actuation of the piezo motors is based on an internal ultrasonic rotor that pushes against the stroke arm at an operating frequency of 166 kHz [35]. The rotor is mounted at a nodal point that minimizes vibrations from the rotor to the environment, and the operating frequency of the motor is high enough that mechanical disturbance is negligible, which preserves the strength of the vacuum attachment. Additionally, reaction forces from needle insertion are not expected to disturb the vacuum attachment as their values range from 0.5–2 N, which is almost half the maximum reaction force allowable as determined by the torque in Equation (1).

The needle drive platform is rigidly fixed to the stroke arm of the motor and has an internal luer fitting to attach commercial needles with integrated OCT sensing to the needle drive platform. The internal luer fitting has a slip fit so that the needle can be rotated to the desired orientation after it has been attached to the internal luer fitting. An internal set screw is used to lock the orientation of the needle, and a barbed fitting on the back of the luer is connected to a syringe so that air can be injected through the needle for pneumodissection of the DM. Three-dimensional front-side and top views of the AutoDALK system are shown in Figure 2b and Figure 2c, respectively. Identified are the key dimensions of the robot including the device width and height, the diameter of the vacuum attachment, and the stroke length of the needle drive platform. The entire AutoDALK system fits within a 25 mm × 40 mm × 30 mm volume and has a mass of 22 g. 

### 3.3. Piezo Actuators

The AutoDALK system uses two XLA-1 piezo actuators (Xeryon, Lueven, Belgium) for linear actuation. Piezo actuators were selected due to their light weight, fast response, and high positional accuracy. The XLA-1 series actuators in particular use ultrasonic vibrations of 166 kHz to expand a piezoceramic stack against a rod driving it forwards or backwards [35]. The XLA-1 actuator has a maximum positional speed of 400 mm/s, stroke length of 15 mm, and no-load positional accuracy of 312 nm when used in a closed-loop configuration with the embedded encoder. Each piezo motor used in this study has a published mass of 5.9 g and generates a maximum of 1 N of axial thrust. While a single motor would be sufficient for needle penetration of the human cornea, two motors are used so that the AutoDALK’s center of mass is aligned above the cornea, and a max thrust of 2 N is achieved for penetration in denser tissues such as ex vivo porcine eyes.

### 3.4. Graphical User Interface and Control

The piezo motors are compatible with Xeryon’s LabView 2021 version 18.0 graphical user interface (GUI) enabling intuitive control of the AutoDALK robot. The GUI supports two independent operation modes for the robot: (1) automatic—the needle moves a predefined depth based on user input; (2) manual—the needle moves a user-defined step size between 312 nm and 100 μm with each button press. The real-time encoder position of the motors is displayed to the user. Communication is performed over USB between the GUI and piezo motors, and the GUI is implemented using Xeryon’s LabView application programming interface (API), which is available for download. Motor speed, operation mode, target depth, and step size are all readily modifiable in the GUI. 

### 3.5. Optical Coherence Tomography (OCT) Design

#### 3.5.1. Common Path Swept Source OCT Imaging Sensor 

**Figure 3:**
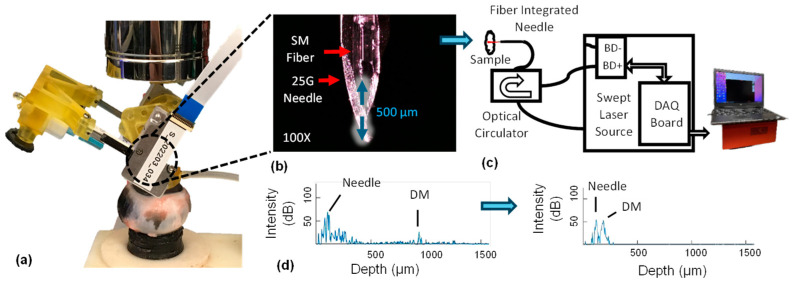
Illustration of the AutoDALK setup for ex vivo testing in porcine eye (**a**), with 100× microscope image of the 25 G needle tip with embedded lensed OCT fiber (**b**). A−scans from the fiber are collected by the common path swept source OCT system illustrated in the block diagram (**c**), and then displayed to the user as a depth map where distance between the needle tip and DM at the beginning (**d**-**left**) and end (**d**-**right**) can be visualized.

The AutoDALK’s real-time depth measurement is acquired by our in-house-built common-path sweep source OCT system (CP-SSOCT) illustrated in Figure 3c [36]. The system’s key components are a single-mode fiber (1060XP, Thorlabs, Newton, NJ, USA), a swept source OEM engine (Excelitas Technologies, Billerica, MA, USA), a broadband circulator (BPICIR-1060-H6, OF-Link, ShenZhen, China), a Camera-Link frame grabber (PCIe-1433, National Instrument, Austin, TX, USA), and a laptop (Precision 5520, DELL, Round Rock, TX, USA). The system operates at a 100 kHz sweeping rate with a center wavelength of 1060 nm, a sweeping range of 100 nm, and an output power of 2 mW. Compared with 2D and 3D OCT systems, our CP-SSOCT system does not need a galvo scanner, which is fully synchronized with the OCT engine, making it compact and easy to integrate with surgical devices. Moreover, it takes the reflected signal from the fiber–ball lens interface as a reference beam, which can remove the influence of polarization and dispersion mismatch [37]. The obtained interferometric signal is processed by a Camera-Link data acquisition board sent to the laptop by the frame grabber. The axial resolution is around 6 µm in air and 4.5 µm in biological samples, with a maximum sensing depth of 3.7 mm and 2.8 mm, respectively.

For this study, the OCT system acquires A-scan signals at a sweeping rate of 100 kHz. This rate is fast enough to provide real-time OCT signals to the user, and the OCT engine outputs 128-buffered spectral data every 1.28 ms. These data undergo high-pass and low-pass filtering to eliminate the DC component and high-frequency noise. Subsequently, zero padding and a fast Fourier transform (FFT) are performed. The 128 A-scan images are averaged into a single A-scan image, with the background signal subtracted. The entire processing sequence is executed by the GPU. The final A-scan image, consisting of 1024 data points, is displayed on the user interface at a rate of 1 kHz, resulting in negligible latency of the system. In this study, the location of the DM is identified by the operator as a peak at the distal end of the A-scan signal (Figure 3d), and the distance between the needle and DM can be manually calculated. 

#### 3.5.2. OCT Distal Sensor Integrated Needle 

The AutoDALK system with an embedded imaging needle setup for ex vivo experiments is shown in Figure 3a, and a microscope view of the OCT-embedded needle design is shown in Figure 3b. The needle assembly includes a commercial 25 G needle with an embedded single mode fiber OCT probe. The inner diameter of the needle (0.260 mm) is slightly larger than the fiber diameter (0.245 mm), which provides clearance for air to flow during pneumodissection. While it is possible to remove the fiber coating so that 27 G and smaller needles can be used, the coating is left on the probes in this study to improve the overall strength of the prototype fiber assembly. To protect the reference plane from the external environment, we fabricate a high-index epoxy half-ball lens (Norland Product, Jamesburg, NJ, USA) at the end of the fiber [38]. The resulting fiber probe has an acceptance angle of 8°, which is large enough to simultaneously detect the needle tip as well as light reflected from objects beyond the needle tip. Before all experiments, the offset between the needle tip and fiber sensor is set to 500 μm using a single-axis translation stage. The fiber is permanently fixed in place by epoxying the fiber to the needle near the needle’s hub. To improve the signal-to-noise ratio of the sensor, the background signal is subtracted from the raw OCT signal during signal collection. An example of the resulting OCT A-scan signal showing depth measurement between the needle tip and DM in porcine corneas is shown in Figure 3d at the start (left) and end (right) of a needle insertion experiment. 

### 3.6. Clinical Workflow

**Figure 4:**
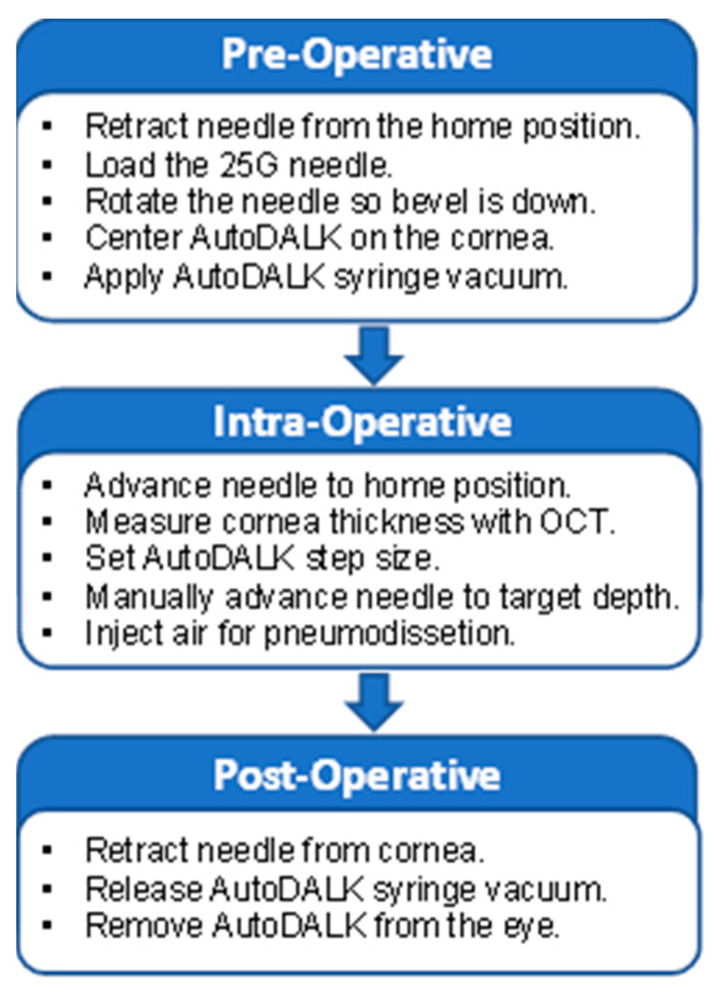
AutoDALK clinical workflow.

The clinical workflow for using the AutoDALK system is highlighted in Figure 4. Using the GUI, the needle drive platform is retracted so that a needle with an embedded OCT fiber can be attached to the luer fitting. The luer fitting is then rotated so that the needle bevel is face down to ensure that the air pressure from the needle is downward and the needle angle is relatively flat to the ground. The orientation is then locked with the set screw. The needle drive platform is then advanced to the zero position, and the AutoDALK system is affixed to the center of the cornea using the vacuum syringe. Prior to the experiments, the system is calibrated by measuring the distance between the fiber probe and needle tip and setting the display pixel size so that this distance matches 500 μm, as expected from the manufacturing of the needle. The distance between the needle tip and the DM is then measured by the operator using the A-scan OCT by calculating the difference between the needle peak and DM peak as illustrated in Figure 3d. Using the GUI described in Section 3.4. The operator defines the needle insertion speed and chooses either a manual operating mode, whereby the robot will move according to a preset step size with each button press, or an automatic operating mode, whereby the robot will move forward to a preset depth that was entered by the user. The user then advances the needle with the GUI using either the manual or automatic modes until the needle has achieved the desired target depth, and air is injected via a syringe through the barbed fitting and needle to pneumodissect the DM. After dissection, the vacuum is removed by depressing the vacuum syringe, and the AutoDALK is removed from the eye.

## 4. Experiments and Results

### 4.1. In Silico Experiments: Finite Element Analysis of the Linear Drive

**Figure 5:**
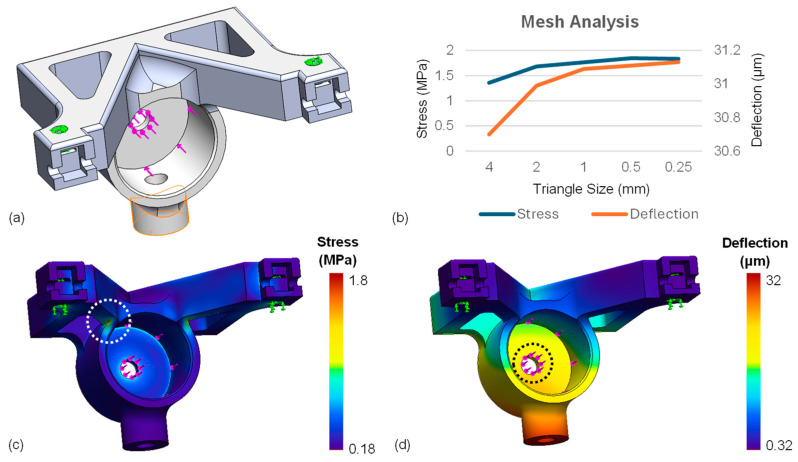
Finite element analysis illustrating fixtures (green arrows) and applied forces (pink arrows) (**a**), mesh analysis (**b**), resulting stress (**c**), and deflection (**d**) with average values calculated from samples in the circled regions.

A finite element analysis (FEA) using the Solidworks Simulation Package 2022 SP5 was designed to ensure that the AutoDALK would perform as expected under maximum loading (Figure 5). When both piezo motors are used for needle insertion, a maximum force of 2 N is imparted on the needle drive platform. For a 616 μm-thick human cornea [39] and a target depth of 80–90% [14,15], the system must be rigid enough to experience less than 60 μm of deflection under a 2 N load so that the DM is not pierced by the needle when the needle is at the full insertion depth. 

#### 4.1.1. Mesh Analysis Study to Determine FEA Parameters

FEA parameters were determined by completing a mesh analysis with a stepwise approach. The mesh analysis was performed using SolidWorks’ design wizard to generate an initial blended-curvature mesh with a maximum triangle size of 4 mm, a triangle growth factor of 1.1, and curvature defined by a minimum of 32 triangles. ABS material with an elastic modulus = 2 GPa, yield strength = 44.36 MPa, and Poisson’s ratio = 0.394 were defined for the part. Virtual fixtures (green arrows) were applied to the contact surface between the needle drive platform and piezo motors, and a uniform force of 2 N (pink arrows) was applied to the contact surface between the needle drive platform and the needle hub (Figure 5a). The maximum stress (defined as the largest observable stress during loading), maximum deflection (largest observable deflection during loading), and minimum factor of safety (FOS, smallest ratio of maximum stress divided by yield strength) were recorded. The analysis was then repeated with half the maximum triangle size until the difference in consecutive stress and deflection simulations was less than 1%. 

Figure 5b illustrates the relationship between the FEA calculated stress and deflection as a function of mesh triangle size. From the graph, the first triangle size that resulted in a difference of less than 1% for both metrics was 0.25 mm. Using a maximum triangle size of 0.25 mm with blended-curvature approach, the final FEA mesh had greater than 1.5 M triangles, 99.9% of triangles had an aspect ratio less than 3, and the mesh had a SolidWorks’ quality rating of ‘high’.

#### 4.1.2. FEA Results 

FEA was performed on the needle drive platform using the mesh derived in Section 4.1.1 to determine the maximum stress, maximum deflection, and minimum FOS of the system. The Results for each metric were obtained by averaging ten random samples taken from the FEA (circled regions in Figure 5c,d). The average maximum stress, maximum displacement, and minimum FOS were calculated to be 1.29 ± 0.086 MPa, 0.026 ± 0.022 mm, and 34.46 ± 2.45, respectively. 

### 4.2. Bench Testing Experiments: AutoDALK Positional Accuracy and Repeatability

ISO 230-2:2014 [40] is an industrial standard that defines a test plan for evaluating single-axis positioning systems. The test method specifies that the system under test be commanded to Five or more target positions $p_{i}$ defined in Equation (2), where *p* is a nominal interval of spacing along the length of travel, *i* is the index of the next target position, and *r* is a random value within ±30% of *p*.

(2) $$p_{i}=\left(i-1\right)p+r$$

From the standard, positional deviation is defined as the difference between the target position and measured position and is recorded when the target is approached from a known starting point in either direction; mean positional deviation is the average positional deviation for all points along the axis in both directions; positional repeatability is four times the maximum standard deviation (σ) of all positional deviations; positional accuracy is the mean positional deviation ±2σ.

**Figure 6:**
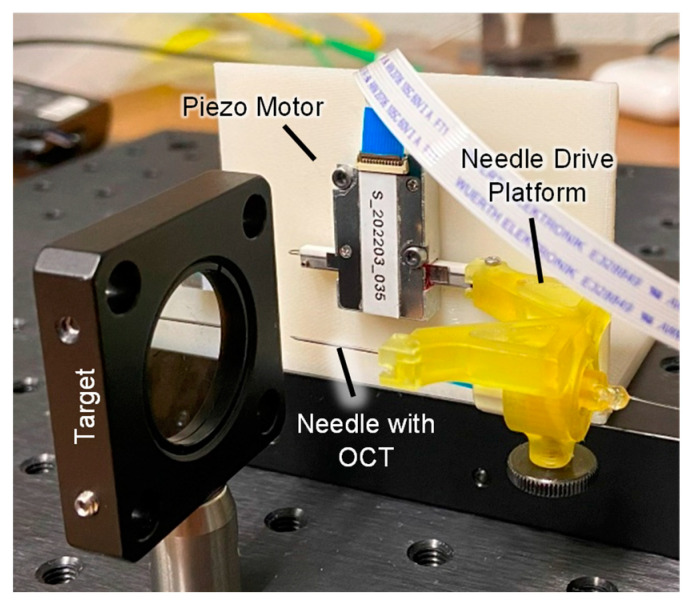
Accuracy and repeatability test setup using a 25 G needle with embedded OCT.

The needle drive platform with the embedded OCT needle was attached to a piezo motor (Figure 6) and the assembly was evaluated for positional accuracy and repeatability according to ISO 230-2:2014. The setup was aligned so that the OCT fiber was perpendicular to a target 2–3 mm away as measured by the OCT fiber. Microsoft Excel version 2405 was used to generate ten positions (p=0.125 mm  in Equation (2)) randomly distributed along a 1.25 mm length of travel. A total travel length of 1.25 mm was chosen because this distance is equivalent to the average stromal thickness (plus two standard deviations) multiplied by a safety factor of 50% to account for stromal swelling in postmortem tissues [39], which will be used in ex vivo experiments. The piezo motor was then commanded to the ten target positions (approaching from both directions), and the distance travelled was measured by the OCT A-scan data. This test was repeated a total of five times for both piezo motors. The mean positional deviation of the system was calculated to be 9.39 µm, the positional repeatability of the system was 39.48 µm, and the positional accuracy was 43.18 µm. The difference in the mean position deviation for both motors was 300 nm across the full travel length of 1.25 mm, resulting in a negligible difference in performance. The result reported is for the worst performing motor as that motor dictated the accuracy and repeatability of the system. The measured mean positional deviation of the system was less than the average thickness of the DM [32], indicating the performance would be sufficient for ex vivo experiments. 

### 4.3. Bench Testing Experiments: Linear Thrust Performance

**Figure 7:**
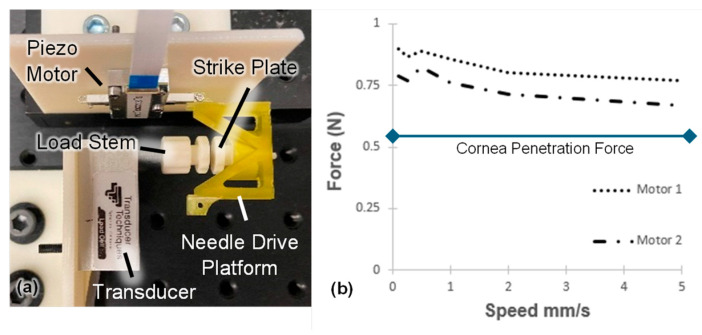
Motor thrust test setup (**a**) and the motor’s resulting maximum thrust profile for different speeds (**b**). The clinical force requirement for cornea penetration is shown with a horizontal line.

The maximum linear thrust of the AutoDALK system was verified using the test setup shown in Figure 7a. In these experiments, each piezo motor was connected to the needle drive platform, which was loaded with a strike plate. The strike plate was then aligned with the load stem of a single-axis load cell (GS0-1K, Transducer Techniques, Temecula, CA, USA). The piezo motor was first advanced with a speed of 0.1 mm/s, and the force against the load stem when the motor stalled was recorded using an NI data acquisition card with a sampling rate of 100 samples/second. The setup was then reset, and the test was repeated using speeds of 0.25 mm/s, 0.5 mm/s, 1 mm/s, 2 mm/s, and 5 mm/s. As illustrated in Figure 7b, the performance of the two piezo motors was similar and had a peak thrust of 0.89 N and 0.83 N, respectively, which exceeds the clinical requirements for the penetration force of corneal tissue [31]. 

### 4.4. Ex Vivo Experiments: AutoDALK versus Freehand Comparison Study

We evaluated the AutoDALK system against an expert surgeon performing freehand needle insertion during a simulated DALK procedure in ex vivo corneas. One-day-old porcine eyes (Animal Technologies, Tyler, TX, USA) were used for this study due to their similarity to human eyes including the presence of the Descemet’s membrane tissue layer, and prevalence as an alternative to human tissue for surgical training [41]. The eyes were pressurized to 20 mmHg to maintain consistency with cornea needle insertion studies described in a previous work [42], by using an injection of saline in the anterior chamber. Prior to all studies, the eyes were imaged with B-scan OCT to measure the starting corneal thickness. 

#### 4.4.1. Experimental Testbeds

**Figure 8:**
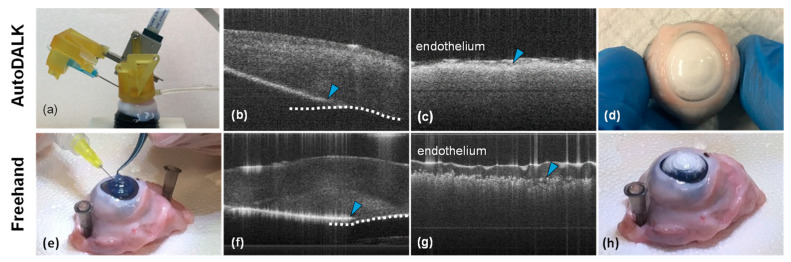
Experimental results for the AutoDALK (**a**–**d**) and freehand (**e**–**h**) approaches. Representative images of the surgical approach (**a**,**e**), final needle depth with an arrow at the needle tip and a dashed line identifying the endothelial layer (**b**,**f**), pneumodissection result with the level of dissection shown by an arrow (**c**,**g**) and endothelium at the top of image, and color image of resulting stromal blanching (**d**,**g**).

Figure 8 illustrates the experimental setup and results of this comparison study. AutoDALK Approach (N = 5, Figure 8a–d): The porcine eye was placed in an ocular holder and fixed using a vacuum syringe. The AutoDALK was fitted with a 25 G needle with an integrated OCT and affixed to the porcine eye using the vacuum channel (Figure 8a). Using A-scan OCT as feedback for the needle depth, a novice operator advanced the AutoDALK system using manual mode until the needle tip was 80–90% the depth of the stroma thickness (Figure 8b). Manual mode was used for all AutoDALK experiments due to soft tissue deformations, which created an unpredictable relationship between the amount of needle advancement and the resulting penetration depth. Air was then injected through the 25 G needle to pneumodissect the stroma (Figure 8c). Freehand Approach (N = 4, Figure 8e–h)*:* The porcine eye was tacked to a Styrofoam board and visualized under a surgical microscope (Figure 8e). Using the standard freehand technique, an expert surgeon inserted a 30 G needle attached to a 5 mL syringe into the porcine cornea and attempted to stop at the depth of the DM (Figure 8f). Air was injected from the 5 mL syringe to pneumodissect the DM (Figure 8g). OCT B-scan images were collected using a separate in-house-built intraoperative OCT system prior to pneumodissection for all eyes so that the needle position at the time of dissection could be calculated. Following the experiments, corneas were removed from the eyes and the endothelial layer was imaged using B-scan OCT to measure the level of the resulting demarcation. The B-mode images were collected using a custom-built SS-OCT intraoperative system, operating at a 100 kHz sweep rate with a 100 nm sweeping range and a center wavelength of 1060 nm. The system provided an axial resolution of approximately 6 μm in air and about 4.5 μm in biological samples, with a lateral resolution of around 12.9 μm. The numerical aperture (NA) of our OCT system was 0.05 [43]. 

**Figure 9:**
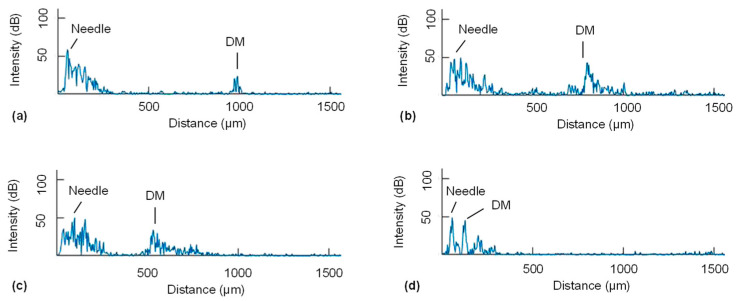
Representative intraoperative OCT A-scan images during ex vivo experiments showing the needle and DM at the start (**a**), middle (**b**,**c**), and end (**d**) of the procedure.

Pneumodissection and stromal demarcation indicating the level of big bubble generation was observed in all five AutoDALK procedures, and 75% of the manual procedures. Representative color images of stromal blanching due to pneumodissection and big bubble generation are shown in Figure 8d,h for the AutoDALK and freehand technique, respectively. Stromal demarcation was not apparent in one of the manual samples due to puncture of the DM. The average thickness of all corneas used in these experiments was measured to be 1071.4 μm. A representative series of images from the A-scan guidance is shown in Figure 9.

#### 4.4.2. Average Needle Depth at Injection

**Figure 10:**
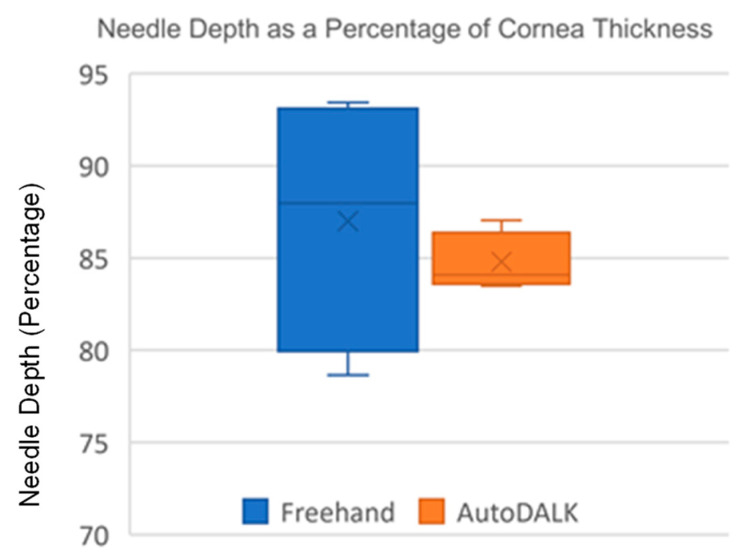
Box-and-whisker plot illustrating the difference in needle depth variance between approaches.

To determine the average needle depth at injection, the endothelial layer was manually segmented from B-mode OCT images taken after the experiments (dotted line in Figure 8b,f). Segmented images were reviewed for accuracy by an expert surgeon who agreed that the segmentations were accurate for all eyes. The needle tip (yellow arrow) was selected, and needle depth was calculated by finding the average 2-D norm from the needle tip to the segmented endothelium. The average depth for each study group was converted to a percentage of total corneal thickness and compared using a two-sample student’s *t*-test with α=0.05. The average needle depth with standard deviation was measured to be 84.81 ± 1.52% and 87.01 ± 6.99% for the AutoDALK and freehand approach, respectively. There were no statistical differences in needle depth between the groups (p=0.189), and both groups were within the 80–90% target range for needle depth [14,15]. Levene’s test for variance was used to check for differences in consistency between the study groups. From this test, the AutoDALK was significantly more consistent than the freehand approach in achieving the target needle depth (p<0.01), which can be visualized by the comparison of the box-and-whisker plots in Figure 10, as well as the comparison of DM perforations using the AutoDALK (N=0) versus the freehand approach (N=1).

#### 4.4.3. Pneumodissection Measurements

Pneumodissection depth was calculated by manually segmenting the stromal demarcation level and endothelial layer (Figure 8c,g) from B-mode images taken immediately after the injection of air. All segmentations were reviewed for accuracy by an expert surgeon. A post-processing program using MATLAB R2022b (MathWorks, Natick, MA, USA) was used to calculate the average distance between the endothelium and demarcation line for each sample, and the distances were converted to a percentage of total corneal thickness. The depth of stromal demarcation was then averaged for each study group and compared using a two-sample student’s *t*-test. The average depth of the stomal demarcation line for the AutoDALK was measured to be 88.69 ± 8.62%, which was significantly deeper than the freehand approach of 70.22 ± 12.57% of the total corneal thickness (p<0.05). There were no statistical differences in consistency of the pneumodissection between study groups (p=0.3177).

## 5. Discussion

This work represents the first known hands-free eye mountable solution for robotic DALK procedures. Using light-weight and geometrically balanced piezo motors with an integrated OCT imaging fiber sensor, we achieved positional accuracy and repeatability of the system, which enabled a more consistent and effective technique for needle insertion and pneumodissection than a freehand surgical approach. The AutoDALK was designed with the clinician in mind, and thus the system was evaluated against clinical requirements. For instance, FEA determined that the system’s factor of safety was 34 times the allowable yield stress, and the part deformation under a maximum load of 2 N was less than 30 µm. This deformation is less than half the target distance between the needle tip and DM during an ideal pneumodissection [14,15,39], indicating that the AutoDALK is not likely to perforate the DM in clinical use. 

Further validation was performed for positional accuracy, repeatability, and maximum thrust generation. Following ISO standards, the AutoDALK demonstrated a mean positional deviation of 9.39 µm, positional repeatability of 39.48 µm, and positional accuracy of 43.18 µm across a total travel length of 1.25 mm. Because the average thickness of the adult DM is 10 µm-thick [32], OCT depth sensing is essential to ensure that the needle advances to the correct position. While the AutoDALK system did not perforate the DM, automatic depth feedback from the OCT signal could be used to further reduce the risk of perforating the DM in future studies. Experiments to measure the system’s maximum thrust also indicated that the performance would exceed the clinical requirements. Either motor’s output exceeds the tissue penetration forces for human (0.518 N) [31] tissue, suggesting that a single motor design may be sufficient for future clinical iterations. 

**Figure 11:**
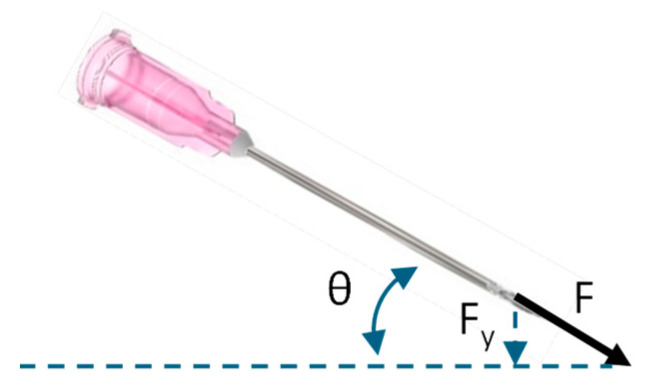
Illustrating the injection angle, θ, injection force, F, and the downward component of the injection force, F_y_, for a needle just prior to pneumodissection.

During ex vivo testing in porcine eyes, the AutoDALK’s position accuracy and OCT depth feedback were essential to achieving a more consistent needle insertion compared to the freehand approach. Ex vivo tissues were chosen for these initial proof-of-concept experiments to demonstrate the safety and efficacy of the system and technique before moving to living tissue experiments. While no statistical difference was found in the average needle depth, the AutoDALK had significantly more consistent needle placement than the freehand approach (*p* < 0.01), which translated into fewer perforations of the DM and the potential for improved visual outcomes. Interestingly, despite both study groups having similar needle depths at injection, the AutoDALK had significantly deeper pneumodissection of the stroma (*p* < 0.05), which is indicative of a better big bubble generation [14]. We hypothesize that the difference in pneumodissection can be attributed to the needle angle during injection, θ, and the downward component of the injection force, F_y_, as shown in Figure 11. For two needles at the same depth, the needle with the larger injection angle, θ (measured from the horizontal), will have a larger downward component of injection force, F_y_, resulting in deeper stromal pneumodissection. The review of the B-mode images found the average needle injection angle during for AutoDALK to be 19.32°, which was larger than the freehand injection angle of 10.90° (*p* < 0.05). Assuming the same injection force, F, between study groups, the downward component of the injection force for the AutoDALK was over 1.75× greater than the freehand approach. One drawback to this increased force is that deep stromal injections may have a higher risk of perforating the DM due to the increased injection pressure. While this phenomenon was not observed in this initial feasibility study, it is possible that perforation may occur in living tissues, or tissues that have a weakening of the corneal layers due to disease. Future in vivo studies will be designed to investigate the relationship between injection angle and the resulting pneumodissection in living tissues to demonstrate the safety and efficacy of the technique. 

Prior to translation from ex vivo to in vivo experiments, however, there are a number of challenges that must be addressed. First, a strategy for sterilization or high-level disinfection will need to be developed to ensure the sterility of the surgical field for survival studies. Due to the sensitivity of the piezo motors to high heat, the system will need to be sterilized using a low-temperature gas such as ethylene oxide or Sterrad. Additionally, the imaging fiber and needle will need to be validated on in vivo tissue to ensure that differences in imaging between ex vivo tissues and in vivo tissues due to postmortem thickening of the stromal layer or swelling of the eye do not result in inaccurate depth measurements of the DM. This will include validating that pneumodissection injections at the 30-degree angle and prescribed depths do not generate an excessive force to perforate living tissue (which may be less stiff than the ex vivo model). Finally, because the gold standard for in vivo cornea experiments is the rabbit model, the AutoDALK’s vacuum trephine will need to be resized to ensure stable attachment to the eye, and that the needle is aligned to the center of the cornea. 

One important clinical outcome that is not addressed in this work is the incidence of type 1 big bubble formation with the AutoDALK system. Type 1 big bubble formation is ideal for the DALK procedure as this type of pneumatic dissection preserves Dua’s layer, which confers additional strength to the recipient cornea. Because the most technically challenging and critical portion of the DALK procedure is deep stromal needle insertion, an ex vivo porcine eye model was chosen for these proof-of-concept studies since the porcine tissue enables a full system evaluation including eye mounting, OCT validation, and the consistency of robotic needle placement, while minimizing the use of human donor tissue. Unfortunately, there are known biomechanical differences between porcine and human tissues [44], which would likely affect pneumodissection and the generation of type 1 big bubbles, a phenomenon that can be observed in deep stromal pneumodissection studies from other groups [45]. We attempt to mitigate this limitation in our studies by providing the pneumodissection depth as an alternative for the incidence of big bubble generation in porcine tissues, as big bubble generation is correlated with deeper pneumodissection [14]. Now that the AutoDALK robot has demonstrated significantly better deep stromal needle placement with corresponding pneumodissection than a freehand approach, the system should be further evaluated using human corneas as in [27] to demonstrate the capability of the system to create more consistent clinical outcomes such as type 1 big bubbles.

An additional limitation of this study is that the AutoDALK needle insertion experiments were performed only under a single intraocular pressure of 20 mmHg. The pressure for this study was selected to be consistent with cornea needle insertion experiments previously published [42]. While normal intraocular pressure can range from 10–21 mmHg [46], keratoconus patients that would be typical candidates for the DALK procedure may have intraocular pressure as low as 14.2 mmHg [47]. To better understand the performance of the AutoDALK system for these patients, we conducted a biomechanical deformation simulation utilizing FEBio [48] to characterize the expected change in deformation of the eye under a typical needle insertion from the DALK system. The simulated eye was modeled using neo-Hookean material properties, with a Young’s modulus of 33,100 Pa and a Poisson’s ratio of 0.42, and the same average geometry as the eyes used in the experiments. For the simulation, the posterior surface of the eye was pressurized to 14 mmHg and 20 mmHg, and a 0.51 N force was applied to the anterior surface of the cornea at the same location as the AutoDALK robot. The results from this study demonstrated that the expected maximum change in stromal deformation for an intraocular pressure of 14 mmHg was only 525 μm. Thus, because the stroke length of the AutoDALK robot is 6 mm, we anticipate the robotic system would still be able to reach the deep stromal tissue with an adequately sharp needle.

It should also be noted that the AutoDALK in this study was designed for use with the average diameter for a porcine cornea, which ranges between 11.0 and 15.5 mm [49]. This size is comparable to the average diameter of a human cornea [50], indicating that the AutoDALK device would be successful at targeting the deep stroma in clinical studies. The AutoDALK’s vacuum channel geometry is purposefully designed such that the robot can be securely eye mounted to a corneal geometry within ±3 standard deviations of the mean. Designing with this requirement means that a single AutoDALK geometry will be compatible with 99.7% of human eyes and follows the same strategy that medical device manufacturers use to market a single vacuum trephine geometry. For cornea geometries that exceed three standard deviations of the human eye, such as rabbit eyes, a new vacuum channel geometry would need to be designed to match the cornea’s curvature. 

With regards to imaging, the results of this study are only possible if the OCT A-scan signal clearly delineates the DM from background noise during needle insertion. Because the magnitude of the OCT signal is directly proportional to the angle of incidence, the AutoDALK system was positioned so that enough signal was detected from the corneal layers. To protect the OCT probe reference surface from the tissue debris and to increase the signal collection angle, an epoxy ball lens was added to the OCT fiber. Alternative studies have indicated that a downward facing OCT probe within a vertical razor edge cannula may be a viable solution to maximize the SNR of the OCT signal by increasing the amount of incident light on the probe [51]. In future studies, we will modify the AutoDALK system to incorporate this vertical edge cannula for robust OCT depth measurements irrespective of tissue irregularity. One point of emphasis, however, is that the performance of the A-scan depth sensor will be relatively unaffected for variations in tissues due to scarring. This is expected because the OCT A-scan depth signal we use in this work is a subset of the OCT B-mode signal, which is used to image clinical cases of corneal scar for multiple disease states including keratoconus [52]. OCT B-mode imaging has already been integrated into clinical microscopes [53], and so the AutoDALK robot would be compatible with any condition that is imaged with these systems. 

Finally, our study demonstrates that the AutoDALK system effectively removes hand tremors, resulting in fewer DM perforations independent of operator experience. Across all tests, a novice user had zero DM perforations using the AutoDALK system, compared to a 25% perforation rate from a surgeon using the freehand technique. A review of the literature suggests that clinical rates of DM puncture remain as high as 11.7% despite years of surgical experience [18]. While this initial experience with the AutoDALK is promising, the proposed system and workflow still require an operator in the loop to interpret the OCT depth signals and set the target depth of the AutoDALK system due to noise that may appear in the A-scan signal when the needle is close to the DM. We chose not to run the AutoDALK system in a closed-loop configuration for these experiments because the automatic detection of the needle and DM for depth calculations must be accurate 100% of the time to ensure that the system does not accidently perforate the DM. We are currently developing a strategy to use signal processing with machine learning techniques to autonomously identify the needle and DM from OCT A-scan data in real time with high accuracy. After registering the OCT fiber probe position relative to the needle tip, a closed-loop feedback loop can be used to zero the distance from the needle tip to Descemet’s membrane to a preset depth of 80–90%, which will be presented in future work. By removing the human from the loop, we hypothesize that this new robotic system will eliminate human variation from sample to sample, resulting in even more consistent needle placement. Additional studies including an in vivo animal model are planned to demonstrate the utility and efficacy of the AutoDALK system to create deep stromal pneumodissection in living tissues.

## 6. Conclusions

To our knowledge, the AutoDALK system is the first hands-free eye mountable robot capable of needle insertion using imaging guidance for the DALK procedure. Initial ex vivo testing demonstrated that the AutoDALK achieves more consistent deep stroma pneumodissection without DM perforation compared to an expert surgeon, which may be beneficial to the DALK procedure. An eye mountable robot has the potential to improve procedure consistency, while simultaneously removing the technical barrier to partial-thickness corneal transplants. Usability and in vivo studies are needed to demonstrate clinical integration of the AutoDALK prior to human testing. In future work, we will explore the use of machine learning techniques to automatically segment the DM in the A-scan signal, which will be used to inform a control loop so that the AutoDALK can be operated in an autonomous mode where the needle reaches the correct depth without user interaction. We will also explore in more detail the relationship between the needle’s angle, injection pressure, and resulting pneumodissection quality to determine the ideal needle depth for big bubble creation while minimizing DM perforations due to injection pressure. Additionally, the system will be evaluated using in vivo experiments including survival studies to demonstrate the safety and efficacy of the robotic technique.

## Figures and Tables

**Table 1:** AutoDALK clinical requirements with associated engineering and performance specifications.

Clinical Requirement	Engineering Specification	Theoretical Performance
Safe Patient Attachment	Patient Mountable	Commercial Vacuum
Small Footprint	<40 mm × 40 mm × 40 mm [30]	24.1 mm × 36.5 mm × 28.4 mm
Light Weight	<35 g (Equation (1))	22 g
Penetrates Cornea	>0.5 N [31]	2 N
High Accuracy	<1 µm [32]	0.312 μm
Insertion Speed	<5 mm/s [33]	10 mm/s
Needle Compatibility	<20 G	22–30 G, 0.5–1” Length

## Data Availability

The original contributions presented in the study are included in the article, further inquiries can be directed to the corresponding author.
